# Harnessing adjuvant-induced epigenetic modulation for enhanced immunity in vaccines and cancer therapy

**DOI:** 10.3389/fimmu.2025.1547213

**Published:** 2025-02-18

**Authors:** Yasmine Megdiche, Rosângela Salerno-Gonçalves

**Affiliations:** Center for Vaccine Development and Global Health, University of Maryland School of Medicine, Baltimore, MD, United States

**Keywords:** adjuvant, epigenetic, vaccine, cancer therapy, immunity, human

## Abstract

Adjuvants are crucial in vaccines and cancer therapies, enhancing therapeutic efficacy through diverse mechanisms. In vaccines, adjuvants are traditionally valued for amplifying immune responses, ensuring robust and long-lasting protection against pathogens. In cancer treatments, adjuvants can boost the effectiveness of chemotherapy or immunotherapy by targeting tumor antigens, rendering cancer cells more vulnerable to treatment. Recent research has uncovered new molecular-level effects of the adjuvants, mainly through epigenetic mechanisms. Epigenetics encompasses heritable modifications in gene expression that do not alter the DNA sequence, impacting processes such as DNA methylation, histone modification, and non-coding RNA expression. These epigenetic changes play a pivotal role in regulating gene activity, influencing immune pathways, and modulating the strength and duration of immune responses. Whether in vaccines or cancer treatments, understanding how adjuvants interact with epigenetic regulators offers significant potential for developing more precise, cell-targeted therapies across various medical fields. This review delves into the evolving role of adjuvants and their interactions with epigenetic mechanisms. It also examines the potential of harnessing epigenetic changes to enhance adjuvant efficacy and explores the novel use of epigenetic inhibitors as adjuvants in therapeutic settings.

## 1 Adjuvants in vaccines

Several types of adjuvants are currently employed to enhance vaccine efficacy through diverse mechanisms and approaches. Historically, aluminum salts, such as aluminum hydroxide and aluminum phosphate, were the most widely used adjuvants. These adjuvants boost the immune response by forming depots at the injection site (*i.e*., depot effect), thereby prolonging antigen exposure. Additionally, they activate the innate immune system by triggering the NOD-like receptor protein 3 (NLRP3) inflammasome pathway, resulting in the production of pro-inflammatory cytokines (1). Though, recent research has shifted toward new compounds with adjuvant properties, including nano- and micro-particles (*e.g*., polymers like poly(lactic-co-glycolic) acid (PLGA) or liposomes), emulsions (*e.g*., MF59 or AS03), immune potentiators (*e.g*., cytokines, Toll-like receptors (TLR) agonists), combination adjuvants (*e.g*., alum with immune potentiators), and mucosal adjuvants to target nasal or oral routes (2). For example, oil-in-water emulsions like MF59 and AS03 enhance local immune activation by promoting antigen uptake by antigen-presenting cells (APCs) and activating the nuclear factor kappa-B (NF-κB) (3). Although traditionally viewed as a pro-inflammatory pathway, recent research has revealed its complexity, suggesting that NF-κB may mediate both pro- and anti-inflammatory responses (4). TLR agonists include a range of molecules such as polyinosinic acid (Poly I) – TLR3 agonist (*e.g*., Investigational use in vaccines for influenza, HIV, and certain cancers) (5), monophosphoryl Lipid A (MPLA) – TLR4 agonist (*e.g*., Cervarix (HPV vaccine) (6, 7), and CpG Oligodeoxynucleotides (CpG-ODN) – TLR9 agonist (*e.g*., Heplisav-B (hepatitis B vaccine) (8, 9). These adjuvants can promote either Th1 immune responses and facilitate the development of CD4+ and CD8+ T cells. Additionally, TLR agonists play a crucial role in modulating Th2 responses by activating B cells and enhancing antibody production against weakly immunogenic antigens (10), thereby improving both the quality and quantity of antigen-specific antibodies.

## 2 Adjuvants in cancer immunotherapies

Beyond traditional vaccines, adjuvants are becoming increasingly important in cancer vaccines and immunotherapies, where they are used to boost the immune system’s ability to recognize and attack tumor cells. Unlike traditional vaccines, which target pathogens, cancer vaccines and immunotherapies must overcome the immune system’s tolerance to self-antigens and its suppression by the tumor microenvironment (TME) (11). One class of adjuvants utilized in cancer vaccines is the stimulator of interferon genes protein (STING) agonists, a class of molecules that activate the STING pathway, a key mediator of inflammation (12). They promote the recruitment of effector immune cells and enhance the priming of tumor-specific CD8+ T cells (13). For example, cyclic GMP-AMP synthase (cGAS)-STING agonists, which activate the cGAS-STING pathway, are linked to the activation of Interferon Regulatory Factor 3 (IRF3) and NF-κB signaling pathways. These agonists promote the secretion of type I interferons, making them promising adjuvants for developing effective subunit vaccines (14). They also detect self-DNA released from tumors or dying cells (15). Suppression of STING signaling through epigenetic silencing impedes DNA damage (16). While commonly used to enhance immune responses in vaccines, saponin-based adjuvants (SBAs), such as QS-21, are also being investigated for cancer immunotherapy. Derived from the bark of the *Quillaja saponaria* tree, SBAs form immune-stimulating complexes that boost antigen uptake by APCs, including dendritic cells (DCs) (17). A recent study examining DC subset responses to SBAs found that the CD163+ CD14+ DC subset is the primary responder to this adjuvant in humans (18). Another class of adjuvants explored for cancer vaccines is the non-nucleoside DNA methyltransferase inhibitor (DNMTi) MC3343, a quinoline-based analog with potential adjuvanticity in osteosarcoma therapy (19). MC3343 reactivates a series of regulatory genes linked to osteoblastic differentiation, which are aberrantly silenced in osteosarcoma, thus helping to restore the balance between cell proliferation and differentiation. Furthermore, MC3343 sensitizes tumor cells to chemotherapy by enhancing the efficacy of doxorubicin and cisplatin. It achieves this by inducing chromatin decondensation, which facilitates drug binding to DNA, leading to increased DNA damage and apoptosis (19). These advancements highlight the role of epigenetic regulation in boosting immune responses.

## 3 Epigenetics

Epigenetics involves inheritable changes in gene expression that occur without alterations to the DNA sequence itself. These modifications can include DNA methylation, histone modification, and non-coding RNA regulation, which collectively influences gene activity and cellular function. Understanding epigenetic mechanisms is crucial for developing better and more effective treatments in the realm of immunotherapies, both for cancer and infectious diseases. DNA methylation was the first epigenetic mechanism recognized. It involves the covalent transfer of a methyl group to the C-5 position of the cytosine ring of DNA by DNA methyltransferases (20). Histones are proteins around which DNA is wrapped, and their chemical modifications can affect how tightly or loosely DNA is packaged. Common histone modifications include acetylation, methylation, phosphorylation, and ubiquitination (21). These modifications can specifically influence the expression of immune-related genes in distinct immune cell subsets (22, 23). In previous studies, mucosal-associated invariant T (MAIT) cells exhibiting distinct cytokine profiles, which were associated with protection against typhoid fever (24), showed infection-induced changes in chromatin marks following *Salmonella enterica* serovar Typhi (S. Typhi) exposure, with these changes being dependent on specific cell subsets (23). Additionally, cross-talk between intestinal epithelial cells and innate lymphocytes, such as natural killer (NK) cells and MAIT cells, played a crucial role in triggering these chromatin modifications within innate lymphocytes (22). Since previous studies have shown that intestinal epithelial cells can differentially recognize closely related strains (25–27), a fundamental question arises: would these epithelial cells induce the same or distinct sets of chromatin changes in response to closely related strains? Non-coding RNAs, including microRNAs (miRNAs) and long-coding RNAs (lncRNAs), can also modulate immune responses by regulating the expression of key immune genes (28). MiRNAs can bind to messenger RNAs (mRNAs) and inhibit their translation or promote their degradation. lncRNAs can interact with chromatin, transcription factors, or other RNAs to regulate gene expression (29). For instance, specific miRNAs have been shown to regulate the differentiation and function of DC and control many aspects of inflammatory processes (30). Exploration of epigenetics pathways in vaccines and immunotherapies allows researchers to design adjuvants that activate immune cells and modify the epigenetic landscape to restore or improve immune function.

## 4 How adjuvants trigger epigenetic changes

**Figure 1:**
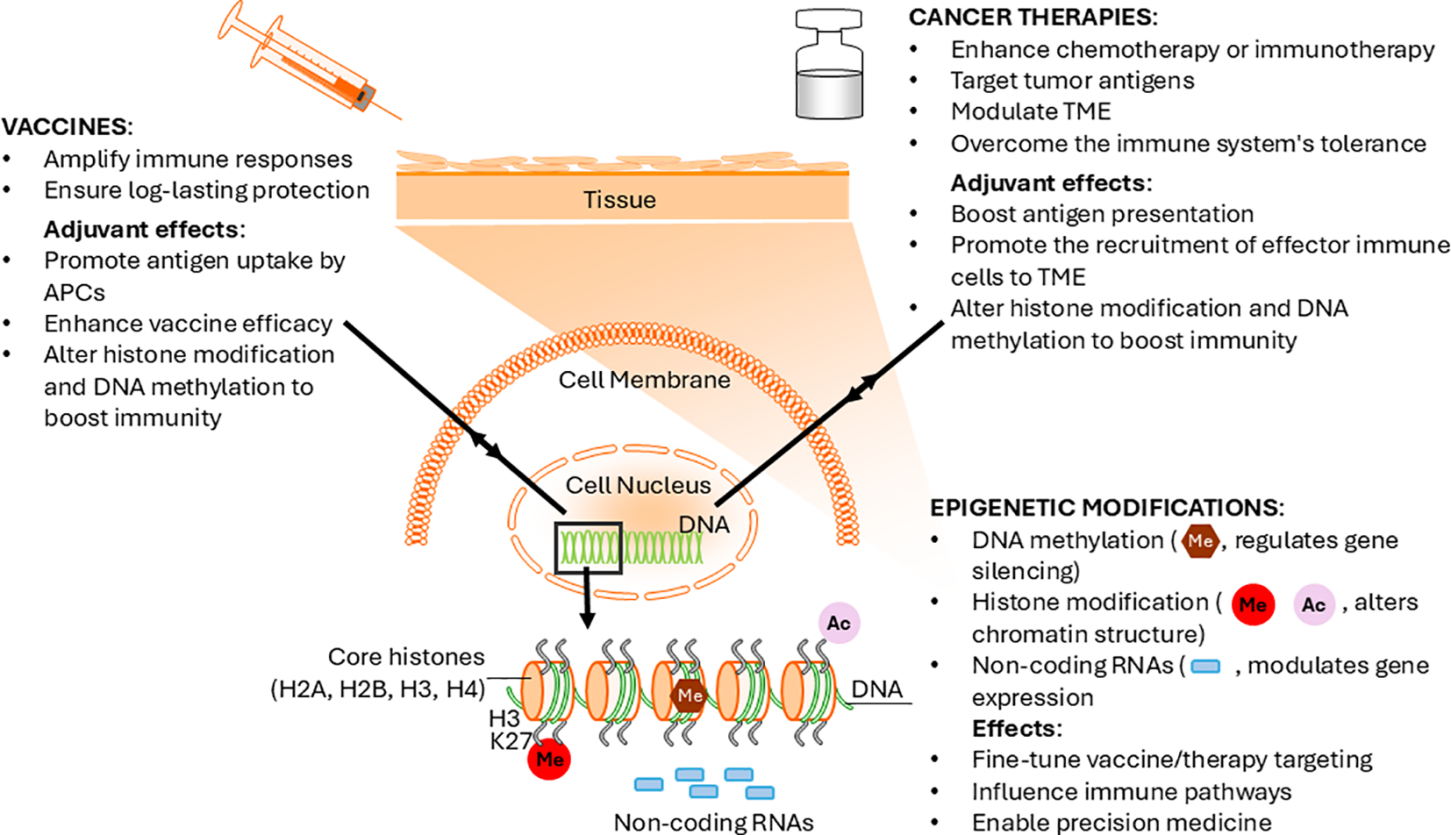
Bidirectional relationship between epigenetic changes and adjuvant potency. Schematic representation of adjuvant-induced epigenetic modulation enhancing immunity in vaccines and cancer therapy.

The signaling pathways activated by adjuvants influence epigenetic changes within immune cells. The elicited modifications lead to alterations in gene expression and immune cell functionality (**Figure 1**). Recent data highlights adjuvants’ ability to reprogram the innate immune system to give rise to heightened resistance against pathogens by training the innate immune system (31). A recent study explored the use of AS03 and its role in epigenetic changes that increased antiviral defenses. Researchers found that including AS03 in the vaccine prompted chemical changes in innate immune cells, leading to increased expression of antiviral genes and resistance to Zika and dengue viruses (32). In contrast, vaccines administered without AS03 failed to induce these epigenetic changes (32). Specifically, AS03 increased chromatin accessibility at loci associated with Interferon Regulatory Factor (IRF) and Signal Transducer and Activator of Transcription (STAT) in monocytes and DCs (33). This improved accessibility facilitated stronger activation of antiviral pathways, whereas non-adjuvanted vaccines resulted in only temporary epigenetic effects. These insights emphasize the dual role of adjuvants in enhancing immediate immune response and in epigenetic reprogramming by altering chromatin accessibility and transcription factor (TF) dynamics to support long-term adaptive immunity. Clinical trials of adjuvants like AS01 and AS03 have shown their ability to enhance adaptive immune responses by activating the IFN-signaling pathway and engaging innate immune cells, particularly innate-like T cells, which undergo epigenetic modifications post-immunization, a phenomenon known as trained immunity (34). Also, yeast-derived adjuvants such as zymosan and β-glucans can stimulate innate immunity and promote trained immunity (35–37). Histone modifications with chromatin reconfiguration have proven to be a central process for trained immunity (38). Novakovic and colleagues showed that β-glucan affects the H3K27ac and H3K4me3 marks in monocytes by reprogramming gene expression patterns during training and upon re-stimulation (36).

In cancer immunotherapies incorporating adjuvants, the epigenetic modifications induced by these agents are crucial, as they enhance the immune system’s capacity to overcome its inherent limitations in recognizing and eliminating tumor cells (**Figure 1**). The interplay between cell types directly involved in tumor lytic activities, such as NK cells, T cells, and macrophages, and the epigenetic regulators plays a crucial role in shaping the effectiveness of cancer vaccines (39, 40). The utilization of adjuvants, such as TLR agonists, induces stable epigenetic imprinting in both normal and cancer cells. There are many TLR adjuvants, each inducing distinct epigenetic changes in immune cells. Stimulation of the TLR3 receptor with Poly I promotes DNA methylation in peripheral blood mononuclear cells (41) and drives the expression of pro-inflammatory cytokines through direct epigenetic regulation at the promoter regions of their gene loci. Additionally, it reactivates silenced miRNAs in tumor cells, thereby enhancing the anti-tumor responses (42). Furthermore, TLR4 ligands can reprogram monocytes by inducing histone modifications that favor the expression of inflammatory genes. These changes have long-lasting effects on monocyte behavior, potentially sustaining an inflammatory response that is beneficial for combating tumor growth (36). While these insights enhance our understanding of the epigenetic changes triggered by adjuvants, significant gaps remain in fully elucidating their precise modes of action, underlying mechanisms, and potential applications in vaccine-based therapies.

## 5 Epigenetic changes modulating adjuvant potency

The interplay between epigenetic changes and adjuvant potency reveals a bidirectional relationship (**Figure 1**). On the one hand, adjuvants induce epigenetic modifications to enhance immune responses. On the other hand, these epigenetic changes modulate the adjuvants’ efficacy. Increasingly, the effectiveness of adjuvants is understood to be closely tied to epigenetic memory, a process associated with trained immunity. Tailoring the epigenetic landscape to maximize adjuvant potency offers promising avenues for developing more effective cancer combinatorial therapies. Advancements in genome-editing tools, such as CRISPR/Cas9, present opportunities for enhancing adjuvant design by precisely manipulating epigenetic marks, leading to an optimized immune response (43). A recent study examining the immune suppressive TME identified HIF1α as a key contributor to the suppressive properties of tumor-associated macrophages. Researchers employed the CRISPR/dCas9-EZH2 system to epigenetically silence HIF1α through targeted histone H3 methylation in its promoter region. This led to sustained repression of HIF α, creating a population of macrophages termed HIF α Epigenetically Repressed Macrophages. When injected into a melanoma mouse model, these macrophages reprogrammed the TME, reducing immune suppression and fostering a tumor-suppressing phenotype. This reprogramming resulted in a notable reduction in tumor burden and an extension of overall survival rates in the treated mice (44). By epigenetically reprogramming immune cells, such as macrophages, to adopt a tumor-suppressing phenotype, we can create a more favorable immune microenvironment that synergizes with adjuvant-enhanced therapeutic models to optimize immune response.

Epigenetic changes can also potentially downregulate inhibitory pathways that suppress immune responses to pathogens, thereby enhancing adjuvant potency. For example, during *S*. Typhi infection, human leukocyte antigen G (HLA-G) expression on infected-target cells significantly contributes to the downregulation of IFN-γ production by MAIT cells (45). This discovery highlights a promising opportunity to epigenetically target the HLA-G pathway to promote robust MAIT cell activation, leading to increased IFN-γ production and a stronger overall immune response. Epigenetic regulation of HLA-G is partly controlled by cis-acting mechanisms (46).

## 6 Epigenetic inhibitors functioning as adjuvants in immunotherapy

Cancer, a disease driven by the accumulation of genetic and epigenetic modifications (47), is increasingly being targeted through therapeutic strategies that modulate the epigenetic landscape and TME. Cancer’s ability to manipulate epigenetic marks allows it to evade immune surveillance and diminish the effectiveness of immune-based therapies. Addressing these cancer-driven epigenetic pathways offers dual benefits: reversing tumor progression and enhancing the efficacy of immunotherapies (48). In epithelial ovarian cancer cell lines that did not express NY-ESO-1, a highly immunogenic tumor-associated antigen capable of eliciting both humoral and cellular immune responses (49), treatment with the DNA methylation inhibitor decitabine (Dacogen^®^) (50) restored its expression, which, combined with a protein vaccine and chemotherapy, led to improved immune responses (51). These findings underscore the potential of epigenetic inhibitory reprogramming to enhance antigen presentation and stimulate stronger immune responses, paving the way for novel cancer immunotherapy strategies. Histone deacetylase inhibitors (HDACis) also function as potent adjuvants by modulating histone acetylation, promoting chromatin relaxation, and enhancing the transcription of immune-related genes (52). For instance, the HDACi AR-42 significantly improved the efficacy of a DNA vaccine targeting the human papillomavirus (HPV) protein E7 in a lung cancer model. This combination resulted in heightened CD8+ T-cell responses and enhanced anti-tumor effects compared to the vaccine alone (53). Epigenetic inhibitors also synergize with immune checkpoint inhibitors (ICIs), making them highly valuable tools in combination with immunotherapies. Enhancer of Zeste Homolog 2 (EZH2), a histone methyltransferase, has been implicated in modulating T-cell activity. In melanoma and bladder cancer models, combining EZH2 inhibitors with anti-cytotoxic T-lymphocyte-associated antigen 4 (CTLA-4) therapy demonstrated a direct role for EZH2-mediated T-cell reprogramming in enhancing anti-tumor immunity (54). CTLA-4 is a critical immune checkpoint that negatively regulates T-cell immune function, and its inhibition enhances immune system activation (55). These findings highlight how epigenetic modulation can act as functional adjuvants, enhancing the efficacy of checkpoint blockade therapies and rendering them effective even in previously resistant tumor types. Beyond enhancing antigen presentation and checkpoint blockade, epigenetic inhibitors broadly modulate the TME. The HDAC inhibitor, 2-hexyl-4-pentylene acid (HPTA), demonstrated significant anti-tumor effects in a rat breast cancer model by leading to the increase in CXCL9/10 mRNA expression and protein levels following treatment (56). CXCL9/10 is involved in immune cell migration, differentiation, and activation (57). This study showed that HPTA treatment enhanced the recruitment of CD4+ T cells to tumor tissues, a process mediated by increased CXCL9/10 expression. The accumulation of T cells at the tumor site boosted the immune response, underscoring the potential of epigenetic inhibitors like HPTA to function as adjuvants in cancer therapeutics.

Additionally, epigenetic modulation has been explored to combat bacterial pathogens, enhancing the body’s natural defenses against infection—an increasingly critical need in light of the global antimicrobial resistance crisis. Drugs targeting epigenetic modifiers of bacterial pathogens, such as methyltransferase inhibitors, hold promising therapeutic potential. For example, current research has focused on *Legionella pneumophila*, the primary causative agent of Legionnaires’ disease, a severe form of acute pneumonia (58). This Gram-negative pathogen utilizes RomA, a SET-domain methyltransferase, to manipulate the host’s epigenetic landscape. RomA facilitates the pathogen’s survival by methylating histone H3 at lysine 14 (H3K14) during infection. To counter this mechanism, researchers developed a high-content imaging screening assay to identify potential RomA inhibitors. These inhibitors offer a novel approach to combating *Legionella pneumophila* by preventing the pathogen from altering host epigenetics (59). Functioning similarly to traditional adjuvants, these inhibitors indirectly enhance the host’s natural defense mechanisms, presenting a promising avenue for anti-infective therapies. The key question, therefore, is: how can epigenetic adjuvants be developed to mimic the potency of live vaccines in inducing robust T-cell responses in humans, which subunit vaccines have thus far failed to achieve, even with potent adjuvants (33)?

## 7 Future directions and conclusions

The dynamic interplay between adjuvants and epigenetic mechanisms holds tremendous promise for paving the path for personalized immunotherapies that lead to better patient outcomes. Many research efforts are underway to explore the potential of these novel tools in immunotherapies. However, significant gaps remain in our understanding of how specific adjuvants influence epigenetic modifications, how epigenetic tools can be used to enhance adjuvant potency, and how these changes, in turn, impact immune responses. Addressing these gaps is crucial for harnessing the full potential of adjuvants in clinical settings. Future studies should focus on elucidating the precise mechanisms by which adjuvants induce their epigenetic changes in immune cells, the duration and stability of these modifications, and their implications for immune memory and response efficacy. By advancing our knowledge in these areas, we can optimize the design of adjuvants and develop more effective, tailored immunotherapy strategies that enhance patient outcomes and combat a wide range of diseases.
